# Gonorrhœal Peritonitis

**Published:** 1887-08

**Authors:** J. E. Greene

**Affiliations:** Mishawaka, Indiana


### GONORRHŒAL PERITONITIS.

BY J. E GREENE, M. D., MISHAWAKA, INDIANA.

[Read before the Mississippi Valley Medical Association in annual session at Crab Apple Springs, Ky., July 12-15, 1887.]

I DOUBT if I could present to your consideration a subject of more importance or less interest than gonorrhœal peritonitis, and in doing so I do not expect to present anything new; but if I can cast a light upon the subject that will cause you to see the gravity and dangers of a simple gonorrhœa I will have accomplished all I hope for.

Gonorrhœa *per se.* is one of our common complaints, and being common is treated as a simple local affection, with too little thought of the possible dangerous *sequelæ.* Even the laity pretend to skill in its treatment; and we as physicians too often say, "but a simple, uncomplicated case of gonorrhœa."

I do not understand how any disease can complicate gonorrhœa, but I can very readily understand how gonorrhœa may complicate many other affections. And while I speak of gonorrhœa simplex (?), in this article it is simply as a preface, and not that I expect to teach you anything in regard to its etiology or treatment.

A secretion procured in non-specific urethritis or vaginitis may be acid in its reaction on litmus. A specific, or gonorrhœal inflammation of any part always causes an acid secretion; the gonococci cannot live in an alkaline menstruum, they travel along a mucous tract rapidly and from choice. I have frequently experimented with gonorrhœal pus, by vaccination in the true skin; and saving to sometimes set up a slight circumscribed dermatitis I have seen no proof that the disease is inoculable on parts of the body other than the mucous tract of the genital organs. You have doubtless seen cases of gonorrhœal ophthalmia, with ulcerated or perforated cornea. The ulcer on the cornea is self-limited, but that limit is not reached until perforation has taken place. I do not mean that perforation is the end wrought by the micrococci, but that the destruction of tissue is so rapid that perforation is merely a mile stone on the road of its action.

Infection of the genital tract is a dangerous affection, and while in the majority of cases "a clap is no worse than a bad cold," no individual ever had the clap that did not have the shadow of a great danger hanging over him.

Gonorrhœal peritonitis presents essentially the same symptoms as a peritonitis from other causes. The symptoms may be more severe, and the progress more rapid, as the cause is more active than many other causes of peritonitis. It is not necessary to give you the symptoms or pathology of peritonitis. You know them too well to mistake them, and see causes too often to mistake them.

I have seen many cases of gonorrhœal epididymitis, and I affirm that not one ever entirely recovered. In 1875 I was called to treat a youth for gonorrhœal epididymitis. He had a mild peritonitis as well. He made an apparent rapid and perfect recovery. In 18[illegible]7 he called upon me for advice, complaining of soreness of the bowels. Examination showed a sub-acute, or rather a chronic inflammation ofthe peritoneum. He told me that since his attack of epididymitis in 1875 he always had a return of abdominal soreness in cold, damp weather, or upon the least excess of any kind.

Another patient contracted gonorrhœa in the summer of 1884. He treated himself, and presumed he was well. Six months after the cessation of the discharge, and three months after any exposure he consulted me. He complained as the previous patient had, of a pain in the abdomen. Examination revealed a tender, slightly swollen testicle. In twelve hours thereafter the testicle was terribly sore and swollen, abdomen tender and somewhat tympanitic, tongue red and dry, temperature 103° He remained in that condition for 48 hours, when there commenced a discharge of pus from the urethra which gradually increased in quantity until it became copious. His epididymitis and peritonitis gradually subsided; he also made an apparent recovery — only apparent, however, for he frequently consults me, always complaining that bad weather causes pain and soreness in the abdomen. I had no means of making a scientific test for gonococci in the pus he discharged per urethram, but I applied some of it to the eye of a dog, and it set up an acute ophthalmia which went on to perforation.

This last case would tend to prove the theory advanced by Noeggareth — which I firmly believe — that gonorrhœa is never entirely cured.

The two cases just cited are in my opinion chronic gonorrhœal peritonitis, and are but types of many others that have fallen under my observation. I have never known a death in the male from peritonitis caused by gonorrhœal infection, although several of our authors cite cases of that kind followed by death.

Whether the germ passes through the vas deferens or semina vesicles to the peritoneum does not matter, either being the high road. In the female the gonococci proceed up through the uterus, through the fallopian tubes to the peritoneum — not in all cases, to be sure, but in many. And while the main symptoms are those pertaining to salpingitis, particularly pyo-salpinx, there is always a peritonitis, circumscribed or general.

In 1879 I was called to treat a lady for puritus vulvæ and leucorrhœa. Examination convinced me that I had a case of gonorrhœa to deal with, and the diagnosis was confirmed by the husband confessing to me that he had had gonorrhœa, and had during the continuance of the discharge, frequent connections with his wife. He also informed me that he had been told by his physician that as his wife was pregnant there was no danger of communicating the disease to her.

Within a few days she miscarried with a six months fœtus. She soon showed symptoms of salpingitis, which was soon followed by a severe attack of peritonitis. Since that time she has been an invalid. At times she is apparently well, often she is confined to her bed with peritoneal inflammation. Refusing any but medical treatment that is far from satisfactory, her condition is one of constant anxiety and danger. Unforeseen and unexpected causes too frequently light the fire of dormant inflammation. It is a sad case, and she is a sufferer indeed.

In 1877 I treated a young married woman for gonorrhœal inflammation of the fallopian tubes and peritoneum.

About a year before marriage her husband had had gonorrhœa, but supposed himself well. He had no urethritis or discharge from the urethra, and she had never had leucorrhœa or vaginitis. This was a case I believe of latent gonorrhœa communicated from the husband. She made an apparent recovery, as they nearly all do, but only yesterday — June 31 — I was summoned to Cincinnati to make a laparotomy upon her, not to save her life, but to relieve terrible and long-continued suffering. I have not yet operated but expect to within the next month.

I do not believe that the gonococci propagate upon the peritoneum, that is a serous membrane, and I do not think it furnishes food enough suitable for the micrococci, but they do propagate within the fallopian tubes, pus, or the abnormal secretion of the tubes which escapes through the fimbria, and thus sets up and keeps up the peritoneal inflammation.

May 1, 1887, I was consulted by a girl aged 19, who complained of extreme soreness in both ovarian regions. She strongly denied having had intercourse, as well as having a vaginal discharge. I suspected gonorrhœa, or that she had had a miscarriage, but she would acknowledge neither. The ovaritis continued to grow worse, and by May 15 she was confined to her bed with peritonitis. She even then maintained her former assertion as to her chastity. By June 4 she was able to sit up, and continued to improve until June 18, when she walked a long distance, and was that evening again compelled to take to her bed. She called no physician until June 23, when I visited her. I found her pulse 160, temperature 105, skin cool and moist, though not cold and clammy, lips blue, *eyes bright;* tongue coated with a brown, moist, thick fur; abdomen tympanitic, and tender, slight cough and dyspnœa. She then acknowledged her exposure, and submitted to a vaginal examination. There was a copious greenish yellow pus discharging from the vagina, the os was inflamed and granular, while from the uterine cavity there was a bloody pus slowly oozing. I was satisfied that the fallopian tubes were filled with pus, also that there was pus in the peritoneal cavity. She was dying from septicæmia. I urged laparotomy for the purpose of removing one cause of blood poisoning, and hoped to be able thereby to save her life. She, as well as the family, objected, and after prescribing I left her. On the morning of June 24 I found her with pulse 120, temperature 101, skin cool and more moist than yesterday, lips slightly redder, tongue cleaner, abdomen tympanitic almost to bursting, countenance very anxious, eyes very bright, and the abdominal pains almost beyond endurance. I prognosticated death within twelve hours, unless she obtained relief. She and her family were anxious for abdominal section, and as a *dernier resort* I consented, though promising nothing. At 2 P. M., assisted by Drs. Thorp and Van Pelt and medical student, Butterworth, I proceeded to operate. After passing through the linea alba, I came upon a thickened and inflamed peritoneum, tense and protruding through the wound already made. I made a puncture through it, when out, spurted with considerable force, pus and serum. I quickly enlarged the incision to about two inches, through which poured pus, serum and broken-down tissue, to the amount, by estimate, of at least three quarts. The odor was horrible. After the evacuation I examined the cavity. I found the fallopian tubes filled with pus and greatly distended; upon the ovaries I found several abscesses from the size of a pea to to the size of a filbert, all filled with pus. The peritoneum was thickened and showed many granular spots. The intestines were distended somewhat with gas, and they, too, in places showed granular spots. Adhesions had formed in many places, but they were not firm. After making as thorough a toilet as possible I closed the wound in the usual manner, leaving in the lower angle a drainage tube. She reacted well, coming soon from under the influence of the anæsthetic, She expressed herself as greatly relieved by the removal of the tension caused by the tympanitis, but I could see she was growing weaker. The skin remained cold although the extremities were warmer, the sweating increased, the lips became a darker blue, the cough and dyspnœa increased. I left her at 8 o'clock P. M. At half-past eleven she died, her mind clear up to the last moment of her life.

I do not think the death was hastened one second by the operation, though I do think that, had I seen her and operated on the 22d, her life would probably have been saved. She died of septicœmia, that might have been controlled if I had earlier removed the cause. The treatment medicinally of gonorrhœal peritonitis differs in no manner from the treatment of peritonitis from other causes, saving perhaps that antiphlogistics are more urgently called for. To my mind surgery offers the most rational mode of treatment, and at the same time promises the most favorable results.

I would not in all cases make a laparotomy, yet whenever the inflammation was active, tympanitis great, and temperature high, I would not hesitate to make an incision through the abdominal walls, clean out the cavity, and insert a drainage tube. In the female I would particularly urge surgical interference, either in acute or chronic cases. If the uterine appendages are affected I would remove them. By so doing the danger to life would not be so materially increased from the operation, and the prospect of complete recovery is too flattering to pass by. Not only would I open the abdomen for gonorrhœal peritonitis, but as well in all cases of severe peritoneal inflammation where I am reasonably sure that there is a serous effusion of dangerous amount within the cavity. I have not yet to regret making the operation, but I do regret not doing so in many cases; I am confident that lives have been lost that might have been saved by timely surgical measures.

In closing, I would particularly urge my professional brethren to consider the gravity of gonorrhœa, and to closely investigate the promises of cure of peritonitis by larapotomy.

